# Towards the 10-year milestone of Journal of Biological Engineering

**DOI:** 10.1186/s13036-016-0038-z

**Published:** 2017-01-13

**Authors:** Jeong-Yeol Yoon

**Affiliations:** Department of Biomedical Engineering and Department of Agricultural & Biosystems Engineering, The University of Arizona, Tucson, AZ 85721 USA

## Abstract

October 10^th^, 2016 marked the 9^th^ anniversary for the Journal of Biological Engineering (JBE), the official journal of Institute of Biological Engineering (IBE), published by BioMed Central. We are entering into the 10^th^ year of its exciting and productive history. In this editorial, a brief history of JBE is summarized, along with a series of analyses on average number of citations, breakdown of topical subjects, geographical representations and so forth for all published articles in JBE. Future prospects and new directions of JBE are also described in this editorial.

## Brief history of Journal of Biological Engineering (JBE)

*Journal of Biological Engineering* (JBE) began its life as the official journal of the Institute of Biological Engineering (IBE; www.ibe.org, headquartered in Lexington, Kentucky, USA). The precursor to JBE was the collection of meeting proceedings (refereed) for the Annual Meetings of IBE. To provide better visibility of those meeting proceedings, numerous discussions were made within IBE to launch its own official academic journal, during early to mid-2000’s. As its first attempt, a select number of presentations from 2005 Annual Meeting of IBE were invited, accordingly peer reviewed, and eventually published in 2006 as the IBE special issue of *Biotechnology Progress* (published jointly by American Chemical Society and American Institute of Chemical Engineers) [1]. This IBE-dedicated special issue was a good success both in terms of quantity and quality, which accelerated the creation of JBE in 2007. Dr. Mark A. Eiteman, the second Editor-in-Chief (EIC) of JBE, has played a pivotal role in contacting a number of publishing houses and negotiating the contract with BioMed Central (BMC; headquartered in London, UK). Once negotiations and the contract were made, BMC became publisher of JBE in 2007. Dr. Mark R. Riley (current department head at University of Nebraska, Lincoln) was named by IBE as the sole Editor-in-Chief (EIC) of this new journal, who also assisted Dr. Eiteman in creating JBE. The first six papers were published on October 10th, 2007 (including Dr. Riley’s introductory editorial [2]), marking the birth date of JBE. At the time of writing this editorial, JBE is slightly over nine years old and is entering into the 10th year of its history. Since then, JBE has served as the one and only official journal of IBE.

The brief history of those 9 years is summarized as follows: Shortly after the launch of JBE, BioMed Central was acquired by Springer Science + Business Media in October 2008 and became a part of Springer. The first EIC, Dr. Mark R. Riley, laid successful foundations having established the journal scope and editorial review process, recruited high-profile Editorial Board Members and Associate Editors, and solicited high-quality submissions over the first 2 years. In late 2008, Dr. Riley expressed his wish to step down from the EIC position. Stepping forward, Dr. Mark A. Eiteman at the University of Georgia (Athens, Georgia, USA) was newly named by IBE as the sole EIC of JBE on January 1^st^, 2009. Under the meticulous and visionary workmanship of Dr. Eiteman the journal continued to grow both in quality and quantity. Specifically, Dr. Eiteman successfully solicited high-profile submissions specifically in the area of synthetic biology, further strengthening the reputation of JBE. Later, it became apparent that additional leadership would be necessary. As a result, I (Jeong-Yeol Yoon) was named alongside Dr. Eiteman as the second EIC of JBE in May 2013 to share the heavy workload.

In May 2015, the manuscript submission system previously developed by BioMed Central was replaced by the Editorial Manager™ (Aries Systems Corporation, North Andover, Massachusetts, USA) for consistency with other Springer journals. Around the same time the JBE journal page was also updated. Later, BioMed Central became a part of Springer Nature following the merger of Springer Science + Business Media and Nature Publishing Group in May 2015.

In late 2015, Dr. Eiteman expressed his wish to step down from the EIC position, and a search to replace him began among the IBE council and the JBE editorial board. Eventually in May 2016, Dr. Raj R. Rao (current department head at University of Arkansas, Fayetteville, Arkansas, USA) was appointed as one of two EICs of JBE. Since then, Dr. Rao and I have recruited new Editorial Board Members to replace outgoing members, and created a new role of Regional Editor. Dr. Sang Yup Lee at Korea Advanced Institute of Science and Technology (KAIST; Daejeon, South Korea) was named the first Regional Editor responsible for the Asia/Pacific area in July 2016. Professor Alfonso Jaramillo (University of Warwick, UK) was named the second Regional Editor responsible for Europe in November 2016.

## Journal impact factor

Since the beginning of JBE, there had been numerous discussions about acquiring the journal Impact Factor from Thomson-Reuters. Some had argued that they would not submit their work to JBE due to the lack of a journal Impact Factor, which would negatively impact their graduation, hiring, promotion, or funding decisions. However, such practice has been warned as a possible misuse of the journal Impact Factor, as addressed in San Francisco Declaration on Research Assessment (DORA) [3].

Nonetheless, it became apparent that there was a need to acquire the journal Impact Factor and to be indexed in the Web of Science™. In turn, this would better represent the overall quality of published papers and allow improvement of content visibility. It should be noted that from the very beginning JBE has had very high visibility: All articles are open access, thus easily searchable and free to download, as well as made immediately available via Google Scholar (scholar.google.com) and PubMed Central (PMC; ncbi.nlm.nih.gov/pmc; by US National Institutes of Health or NIH).

The approach taken by the JBE was rather a conservative but solid one. Instead of inviting and accepting large bulk of potentially low quality papers, or soliciting large number of review papers that would invalidly boost up the journal Impact Factor, JBE has strived to focus on very high quality research papers. In fact, the “unofficial” Impact Factor of JBE calculated by BioMed Central rose to 3.91 in the year 2012, one of the very highest in engineering disciplines. However, this conservative policy has inevitably led to the low number of published papers, preventing Thomson-Reuters to start their tracking of JBE’s journal Impact Factor.

Fortunately, at the beginning of 2012, Thomson-Reuters started indexing JBE articles in their Web of Science™ database, signaling the tracking of journal Impact Factor. The first “official” journal Impact Factor was announced by Thomson-Reuters as 2.481 for the year 2014 (released in summer 2015). It later boosted to 3.000 in 2015 (released in summer 2016).

Thomson-Reuters also provides the rank of journals. For reasons unknown, Thomson-Reuters classifies JBE in “Biochemical Research Methods” (27^th^ out of 77 journals) and “Biotechnology & Applied Microbiology” (49^th^ out of 161 journals), but not in any engineering disciplines, such as “Biomedical Engineering,” “Chemical Engineering,” or “Multidisciplinary Engineering.” We believe this is a misinterpretation, since JBE rarely publishes papers about biochemical research methods (a good example can be found in method-specific journals, for example, *Nature Methods*) or microbiology science subjects. If JBE were listed under Biomedical Engineering, it would rank 15^th^ out of 77 journals with Impact Factors (81.2% percentile), just above *Annals of Biomedical Engineering*. For Chemical Engineering, it would be 23^rd^ out of 136 journals with Impact Factors (83.5% percentile), just above *AIChE Journal*. For Multidisciplinary Engineering, it would be 8^th^ out of 86 journals with Impact Factors (91.3% percentile). If properly ranked, JBE would be considered as one of the top-tier journals in engineering disciplines in terms of the journal Impact Factor.

## Topical areas of JBE

In Dr. Riley’s introductory editorial [2], the following five topical areas wereidentified:

- Synthetic biology and cellular design
- Engineering of devices that interface at the biomolecular, cellular, and organismal levels
- Bioproduction and bioproduct engineering
- Ecological and environmental engineering, and
- Biological Engineering education and the biodesign process.

These topical areas roughly correspond to:

- Synthetic biology
- Biomaterial (including drug delivery and tissue engineering)
- Bioproduction (including metabolic engineering)
- Biosensor
- Environmental engineering (including ecological engineering)
- Biological engineering education (including biodesign process)

**Fig. 1:**
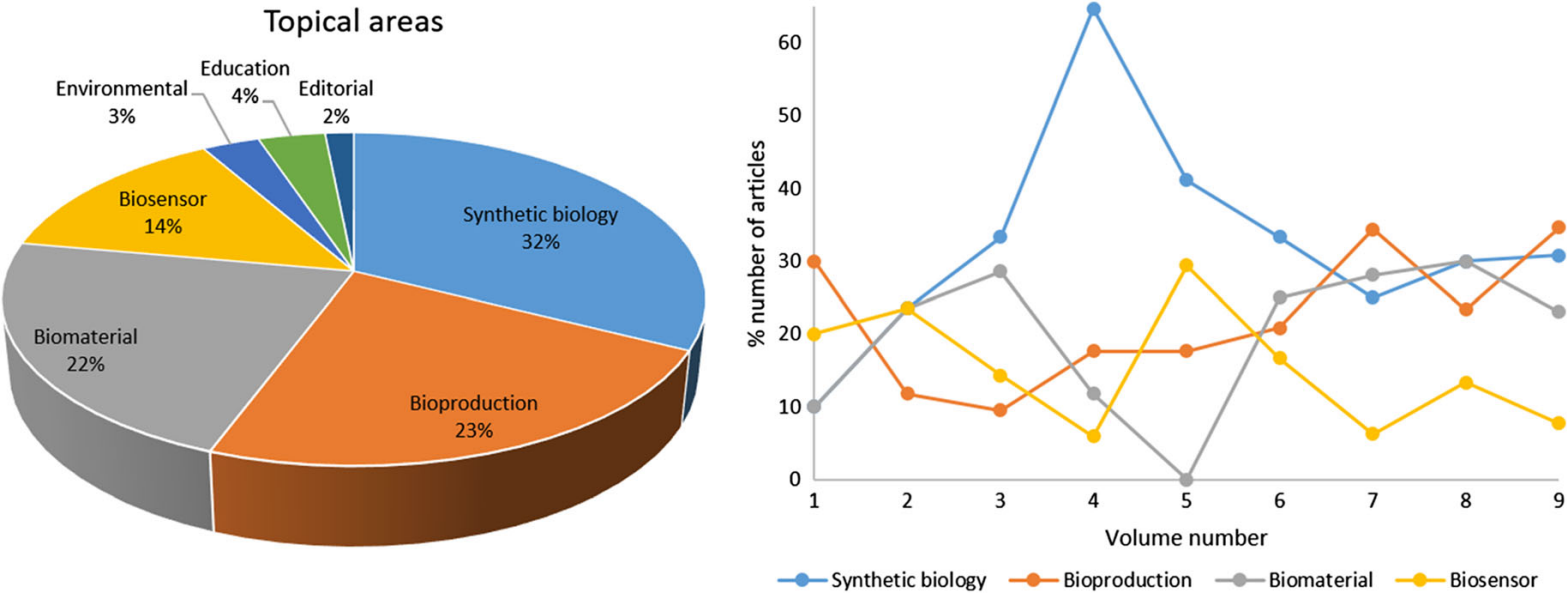
Topical areas of the published JBE articles from volumes 1 to 9 (2007–2015): all papers (*left*) and papers by volume (*right*)

Whilst biosensor was not specified in [2], I have included it as a major topic since JBE has published a substantial number of papers on biosensors. I have classified all published JBE articles (from volumes 1 to 9, i.e. years 2007 to 2015; note that JBE publishes one volume per year) into the above six areas, and summarized the results in Fig. 1. I have to admit that such classification was not always easy, since biological engineering is inherently interdisciplinary. It was also difficult to draw a clear borderline between synthetic biology and bioproduction. While I tried my best to make such classifications, there may exist a certain degree of misinterpretation. Also in some rare cases, two different topical areas were equally addressed in a single paper, thus I counted such paper twice over two topical areas.

As shown in Fig. 1 in the left, synthetic biology makes up 32% of published JBE articles, followed by bioproduction (23%), biomaterial (22%), biosensor (14%), environmental engineering (3%), and education (4%). As shown in Fig. 1 in the right, a substantial spike in synthetic biology in volume 4 (year 2010) occurred, which led to the highest “unofficial” Impact Factor in 2012 (3.91). (The 2012 Impact Factor is calculated by the number of citations across Web of Science™ database made in 2012 that cite JBE papers published in 2010 and 2011). The decline in synthetic biology after volume 5 (year 2011) can be associated with the launch of other synthetic biology-specific journals, most notably *ACS Synthetic Biology*, which began publishing accepted articles in the fall of 2011. In the meantime, the number of bioproduction submissions has steadily increased over the years. The number of biomaterial submissions however, initially started strong, plummeted through volumes 4 and 5 (years 2010 and 2011). Fortunately, healthy numbers returned post volume 6 (year 2012), partly due to the thematic series titled “Emerging biomaterials for translational medicine,” led and edited by me. Throughout volumes 7, 8 and 9 (years 2013, 2014 and 2015), synthetic biology, bioproduction, and biomaterial papers were roughly equal in their numbers of published articles, thus comprising the three major topical areas of JBE.

While the number of biosensor submissions was initially healthy, it has gone through a slight decline since volume 6 (year 2012). To address this issue, I have recently initiated another thematic series titled “Biosensor Technologies,” which is scheduled to be published in early 2017. In addition, Dr. Raj R. Rao, another EIC of JBE, has also initiated a third thematic series entitled “Stem Cell Engineering and Regenerative Medicine.” While JBE has published substantial number of biomaterials, drug delivery and tissue engineering articles, stem cell engineering and regenerative medicine have not been major foci of JBE in the past. We believe this new thematic series will further strengthen the existing scope of JBE and draw significant interest from biological engineering research communities.

Since 2008, JBE has announced one “Publication of the Year” award per year based on the highest number of downloads in that given year. These awards have been recognized and subsequently awarded in Annual Meetings of IBE, typically held in March every year. Table 1 summarizes the results. While the previous awards have been made primarily to synthetic biology papers, recent awards were diversified to include bioproduction and biomaterial papers.

**Table 1:** List of JBE Publication of Year awards

Year	Authors	Title	Article No.	Topical area
2008	Haynes KA et al.	Engineering bacteria to solve the Burnt Pancake Problem	2:8 [5]	Synthetic biology
2009	Baumgarder J et al.	Solving a Hamiltonian Path Problem with a bacterial computer	3:11 [6]	Synthetic biology
2010	Agapakis CM, Ducat DC, Boyle PM, Wintermute EH, Way JC, Silver PA	Insulation of a synthetic hydrogen metabolism circuit in bacteria	4:3 [7]	Synthetic biology
2011	Shetty RP, Endy D, Knight TF	Engineering BioBrick vectors from BioBrick parts	2:5 [8]	Synthetic biology
2012	Ortiz ME, Endy D	Engineered cell-cell communication via DNA messaging	6:16 [9]	Synthetic biology
2013	Kahl LJ, Endy D	A survey of enabling technologies in synthetic biology	7:13 [10]	Synthetic biology
2014	Latterman C, Funke M, Hansen S, Diederichs S, Büchs J	Cross-section perimeter is a suitable parameter to describe the effects of different baffle geometries in shaken microtiter plates	8:18 [11]	Bioproduction
2015	Chia HN, Wu B	Recent advances in 3D printing of biomaterials	9:4 [12]	Biomaterial

## Geographical representations

**Fig. 2:**
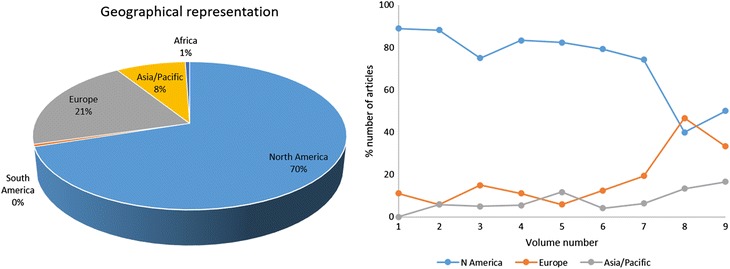
Geographical locations of the corresponding author’s affiliated institutions for the published JBE articles from volumes 1 to 9 (2007–2015): all papers (*left*) and papers by volume (*right*)

I have also identified the geographical representations of the corresponding author(s) (i.e., the geographical locations of their affiliated institutions) of all published JBE articles, again from volumes 1 to 9, i.e. years 2007 to 2015. If two corresponding authors from two different continents were encountered for a single paper, they were counted twice over two continents. Figure 2 shows the results.

Since JBE began its life as the official journal of IBE, which was essentially a USA-based academic institution, most papers from volume 1 (year 2007) to volume 6 (year 2012) were originated from North America, almost exclusively from USA. Since volume 7 (year 2013), however, JBE expanded its editorial board to include more members from Asia/Pacific and Europe, and as such, its geographical representation has greatly diversified. In volumes 8 and 9 (years 2014 and 2015), contributions from North America and Europe are comparable while those from Asia/Pacific have steadily increased.

**Fig. 3:**
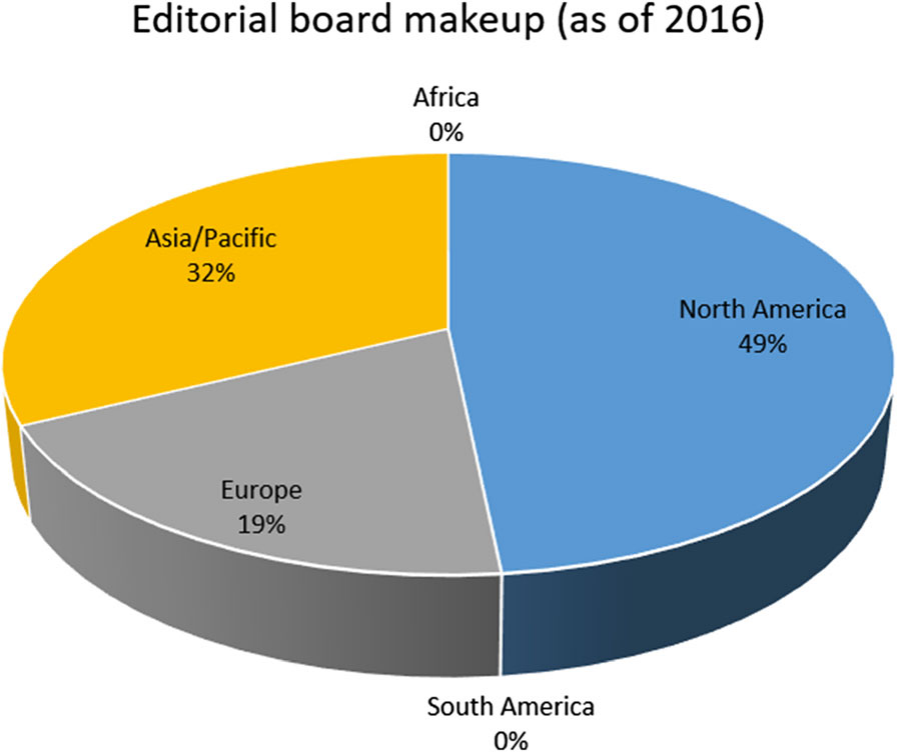
The current makeup of JBE editorial board, as of November 28^th^, 2016, broken down by continents

Figure 3 shows the current break down by associated continent of the JBE editorial board, including current and past EICs, Regional Editors, Associate Editors, and all Editorial Board Members. Compared to the initial makeup, diversity has increased significantly, with North America representing less than a half.

As explained in the brief history section, JBE has named its first Regional Editor in July 2016, Dr. Sang Yup Lee from KAIST (Daejeon, South Korea), to better represent Asia/Pacific region. The second Regional Editor responsible in representing the European area is Dr. Alfonso Jaramillo (University of Warwick, UK), announced in November 2016.

## Article types and review time

JBE publishes the following article types:

- Research
- Letters to the Editor
- Methodology
- Review (including Book Review)
- Editorial (including Meeting Report)

**Fig. 4:**
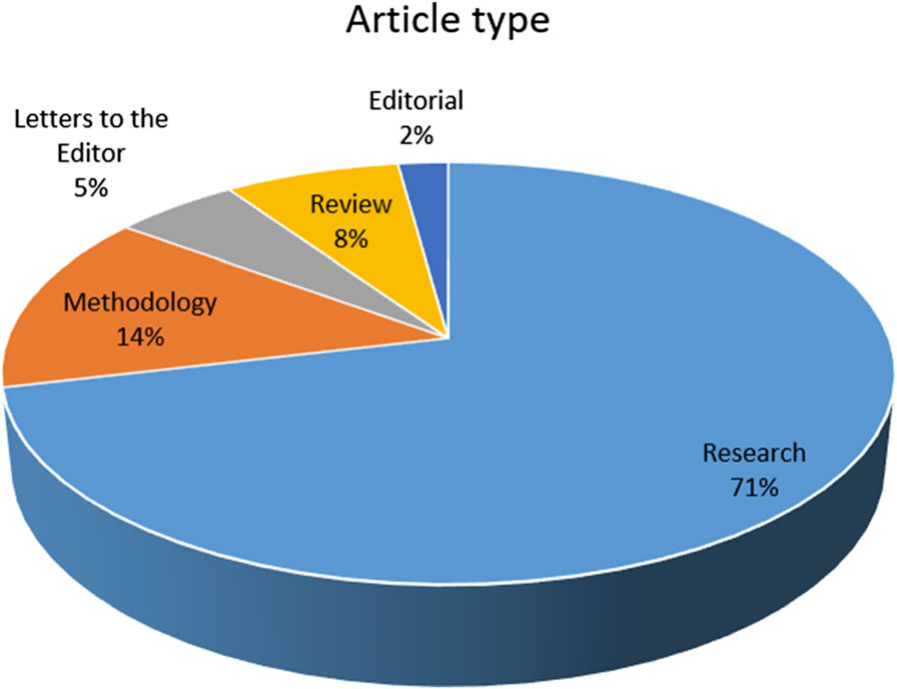
The makeup of all published JBE articles based on their type, from volumes 1 to 9 (2007–2015)

Figure 4 shows the makeup of all published JBE articles based on their types. The majority of published papers were Research (71%), followed by Methodology (14%) and Letters to the Editor (5%). Review papers makes up only 8% of published articles.

The time to reach the first decision, or days taken from initial submission to the first decision, has steadily decreased from 58 days in 2009 to 34 days in 2016, through making concerted efforts in reducing this time.

## Where JBE stands now, and What about Future Opportunities?

Biological engineering is widely considered as a very unique engineering discipline, in terms of its breadth and multidisciplinary character. It may embrace other well-established engineering disciplines such as biomedical engineering, chemical engineering, environmental engineering, and agricultural engineering under its umbrella. However, what makes biological engineering unique is not just its inclusive nature, but rather its unique potential to connect these different engineering disciplines or even to connect other applied science disciplines to converge into a whole new research theme, technology, or discipline. Specifically, a system-wide approach typically adopted in biological engineering, often converges entire ecological systems, making the biological engineering discipline substantially broad-based and inter-/multidisciplinary. These unique characteristics should undoubtedly provide exciting academic as well as industrial opportunities.

Such emerging biological engineering disciplines have been previously addressed in the JBE article jointly written by myself and Dr. Riley [4], in conjunction with 14 grand challenges addressed by U.S. National Academy of Engineering. Specific examples of such emerging inter-/multi-disciplinary biological engineering may include:

- Synthetic biology specifically with engineering design and prototyping emphasis, towards producing commercially viable bioproducts,
- Personalized medicine based on innovative biomaterials, drug/gene delivery and tissue engineering technology,
- Development of field-ready and handheld biosensors, utilizing recent advancements in optics, smartphone technologies, wearable devices, and big data approaches,
- Wearables, internet of things (IoT), and mobile health,
- Ecological system-wide approach for wastewater treatment, water recycling/reuse, monitoring environmental toxicants, and sustainable production of natural products,
- Robotics that can be used for precision agriculture as well as automated laboratory diagnostics, and
- Ecological system- and big data-based approaches in proteomics and metagenomics.

This list is not exhaustive and many other exciting themes may emerge in the future. Altogether, these new opportunities may lie under the broad umbrella of biological engineering, where JBE can provide an exciting discussion forum.

## Concluding remarks

As summarized in this editorial, JBE has witnessed an exciting history of its growth over the past 9 years. Overall quality of submissions has improved with solid journal Impact Factor, the number of published papers has been rising steadily, and the geographical representations of both published papers and editorial board has diversified, while focusing primarily on solid research articles. The broad-based and inter-/multidisciplinary characteristics of JBE have obviously enabled its strong position and reputation across engineering journals, although there still exists more room for future growth. In addition, this unique, broad-based engineering discussion platform is made publicly available through its open-access platform. We will continue to strive for maintaining high-quality and providing real impacts to biological engineering communities throughout the world. As such, we strongly encourage you to consider submitting your next work to this exciting engineering platform.
